# Exercise Is Medicine…and the Dose Matters

**DOI:** 10.3389/fphys.2021.660818

**Published:** 2021-05-12

**Authors:** Sean P. Langan, Gregory J. Grosicki

**Affiliations:** ^1^Department of Kinesiology, Korey Stringer Institute, University of Connecticut, Storrs, CT, United States; ^2^Department of Health Sciences and Kinesiology, Biodynamics and Human Performance Center, Georgia Southern University (Armstrong Campus), Savannah, GA, United States

**Keywords:** metabolism, exercise prescription, mitochondria, cardiovascular, exercise physiology, human performance

## Introduction

The cellular events underpinning exercise adaptation have long been studied since Holloszy's novel findings in rodents over 50 years ago (Holloszy, 1967). Contemporary advances in laboratory techniques have allowed researchers to further explore physiological adaptations to physical activity, and much attention has been given to elucidating the distinct biochemical responses to exercise of varying intensities. More specifically, comparison of high intensity interval training (HIIT) or sprint interval training (SIT), to moderate intensity continuous training (MICT) has been an area of great interest among exercise physiologists. Spearheading these investigations, Burgomaster and colleagues provided evidence for robust and comparable metabolic adaptations when 6-weeks of low-volume SIT was pitted against conventional MICT, despite markedly reduced total training volume in the SIT group (66% less time, 90% less energy expenditure) (Burgomaster et al., 2008). Follow-up studies supporting these findings have provided continued enthusiasm for HIIT/SIT as a time-efficient strategy to improve cardiometabolic health (Gillen and Gibala, 2014; Gillen et al., 2016; Wolfe et al., 2020). HIIT has since become one of the most popular fitness trends as reported by the American College of Sports Medicine (ACSM) (#5 in 2021 survey) (Thompson, 2021). While we acknowledge these findings and are advocates for HIIT, we feel as though it is also important to appreciate the discrepant nature of HIIT and MICT, which may foster unique physiological adaptations with relevant health implications. Few, if any of these adaptations are mutually exclusive, but their magnitude may vary in a manner that should be considered to when determining exercise prescription.

## Cardiovascular Implications and Diminishing Returns

Cardiorespiratory fitness (i.e., VO_2_max) is an independent predictor of cardiovascular and all-cause mortality (Myers et al., 2002) and sets the upper limit for aerobic metabolism (Joyner, 1991; Joyner and Coyle, 2008), justifying its use as an endpoint for studies comparing HIIT vs. MICT. By in large, most (Burgomaster et al., 2008), but not all (Gillen et al., 2016; Gerosa-Neto et al., 2019), studies show greater improvements in VO_2_max with HIIT vs. MICT (Milanović et al., 2015) (Helgerud et al., 2007). For example, in an 8-week training study Helgerud and colleagues observed significantly greater improvements in VO_2_max (~4-5ml/kg/min), cardiac output (~3L/min), and stroke volume (~15ml/beat) with HIIT [~95% max heart rate (MHR)] vs. MICT (70% MHR) (Helgerud et al., 2007). Similar findings were seen in obese men and women where the 4 × 4-min group had greater VO_2_max gains (10%) compared to 10 × 1-min (3.3%) or 45-min of MICT (3.1%) (Bækkerud et al., 2016), but differs from the results of Morales-Palomo using equivalent exercise dosages in those with metabolic syndrome (MetS), where all groups improved equally (~12%) (Morales-Palomo et al., 2019). However, given the documented role of central factors in limiting maximal oxygen consumption (Saltin, 1985), it seems reasonable that efforts limited by VO_2_max would preferentially promote central cardiovascular adaptations. An important consideration is the potential for diminishing returns (Tjønna et al., 2013) and cardiac complications (O'keefe et al., 2012) with excess volumes of high intensity endurance training. In untrained but otherwise healthy subjects, Tjonna and colleagues demonstrated that a single 4-min high intensity treadmill bout 3x/week elicited equivalent improvement in VO_2_max (~10%) as the same workload repeated four times per session (Tjønna et al., 2013). Further, it should be noted that extreme high intensity training volumes undertaken in a small subset of endurance athletes may be deleterious to heart health (O'keefe et al., 2012). Nonetheless, the inverse dose-response relationship between exercise volume and mortality (Arem et al., 2015) suggests volume plays a critical role in mediating the health benefits associated with physical activity, but unfortunately the specific dose of exercise was unavailable in this retrospective cohort. Ultimately, whether the benefits of MICT can be achieved through low volume HIIT is unclear, and worthy of greater scrutiny.

## Peripheral Metabolic Adaptations and Complex I Bypass

Prolonged efforts of low-moderate intensity training (40–70% HRR; <lactate threshold) provide a potent stimulus for peripheral adaptation, and specifically maximal mitochondrial respiration. Lower metabolic byproduct accumulation during this type of activity allows for substantially greater exercise duration and reliance on oxidative energy production and mitochondria-derived ATP, with repeated efforts effectively training optimal substrate efficiency (greater phosphorus:oxygen ratio; P/O). Indeed, Bækerrud et al. showed a greater increase in work economy for MICT when compared to HIIT (Bækkerud et al., 2016). In 2019, Nilsson and colleagues used *in silico* modeling to examine optimal metabolic pathways for ATP synthesis (Nilsson et al., 2019). They discovered that beyond ~40% VO_2_max, mitochondrial energy flux utilizes the glycerol-phosphate shuttle (Green, 1936; Mráček et al., 2013) to transport electrons directly to ubiquinone and entering at complex III, effectively bypassing complex I to avoid to “backpressure” of the complex I proton pump (Nilsson et al., 2019; Glancy et al., 2020). Notably, this is nearly the same workload where Vollestad reported that type II fibers recruitment begins (Vøllestad and Blom, 1985), which are less reliant on oxidative energy production and have greater state 3 respiration with glycerophosphate as a substrate (Willis and Jackman, 1994). While this model rested on various assumptions, it was consistent with world record running speeds and human data, in which the model was unable to match human gas exchange without complex I bypass. However, the increased catalytic rate for ATP generation is compensated for by sacrificing substrate efficiency via reduction of the inner membrane proton gradient. Nilsson et al. showed that the various energy pathways display Pareto optimization, where complex I bypass demonstrates a balance between complete beta-oxidation (efficient) and fermentation (catalytic) (Nilsson et al., 2019). The threshold at which the glycerol phosphate shuttle begins to preferentially bypass complex I (~40% VO_2_max) was thus coined “Complex I max” (CI_max_), which is characterized by a disproportionate increase in oxygen consumption reflecting reductions in mitochondrial efficiency owing to complex I bypass.

If an individual were to participate in exclusively HIIT, it is reasonable to speculate they could develop adaptations that would render them less substrate efficient by consistent bypass of complex I (decreased CI_max_). To this end, Gibala et al. noted that using SIT as a means to enhance prolonged endurance is not recommended (Gibala et al., 2006). It is tempting to suggest that as a result of the inverse relationship between lactate and fat oxidation (San-Millán and Brooks, 2018), excessive HIIT and concomitant lactate accumulation performed chronically could hinder optimizing fat oxidation. While some HIIT vs. MICT studies show similar acute molecular signaling and enzyme adaptations (Gibala et al., 2006; Burgomaster et al., 2008), earlier work has suggested that mitochondrial enzymes alone are not indicative of exercise metabolism or endurance performance in highly trained athletes (Coyle et al., 1988). As reported in a review by MacInnis and Gibala, the similar responses are likely attributed to greater metabolic stress via increased ATP turnover, reactive oxygen species (ROS), Ca2+-calmodulin-dependent protein kinase II (CaMKII) signaling, and intracellular metabolite accumulation (lactate, AMP, ADP) in HIIT trials that may compensate for reduced training volume during MICT (Macinnis and Gibala, 2017). It is important to note that diminishing returns with SIT have also been demonstrated at the level of the skeletal muscle. Parolin et al. provide evidence for inhibition of glycogenolysis (decreased phosphorylase) and reduced glycolytic metabolite production after three vs. one Wingate bout (Parolin et al., 1999), suggesting that SIT responses may be downregulated by repeated bouts.

Interestingly, Coyle and colleagues work on two groups of competitive cyclists matched for fitness and with similar mitochondrial enzyme activity (citrate synthase and β-hydroxyacyl-CoA dehydrogenase) demonstrated markedly different time-to-exhaustion performance, post-exercise lactate, % VO_2_max at lactate threshold, and capillary density (Coyle et al., 1988). Both of the aforementioned enzymes have been previously measured to compare metabolic adaptations between HIIT and MICT (Burgomaster et al., 2008). While relevant, using these enzymatic markers in isolation as outcome variables to substantiate similar metabolic adaptations between HIIT/SIT and MICT may not provide an all-encompassing view of metabolic adaptation (i.e., NADH shuttles), as evidenced by the discordant lactate levels between groups (2-fold difference) after cycling to exhaustion at 88% VO_2_max (Coyle et al., 1988). Thus, if mitochondrial function were truly similar, one would expect handling of lactate to follow suit, since lactate metabolism is “intimately tied to mitochondrial function and volume density,” as reported by Glancy et al. (2020). The importance of standardization and interpretation when measuring mitochondrial content and respiratory function has been highlighted in an elegant review by Bishop et al. (2019a). While other mechanisms such as capillary density likely contribute to the apparent metabolic differences between groups, it would be interesting to evaluate the role of CI_max_. Although this idea is speculative and has not been directly examined, it fits with many of the peripheral adaptations seen with frequent prolonged low-intensity training (i.e., did the higher performing group have a greater CI_max_?). In Flockhart and Larsen's recent commentary regarding the highest VO_2_max ever reported, they noted the following regarding physiologic efficiency: “…very little is known which metabolic factors and muscle characteristics that govern efficiency, or which type of training is responsible for long-term decreases or increases in efficiency” (Flockhart and Larsen, 2019). As co-authors on the Nilsson manuscript, one must wonder if they were hinting at their complex I bypass findings to be published 2 months later, which might suggest that prolonged low intensity efforts near CI_max_ may be warranted for improving substrate efficiency.

## Comparison Between HIIT/SIT and MICT

**Figure 1:**
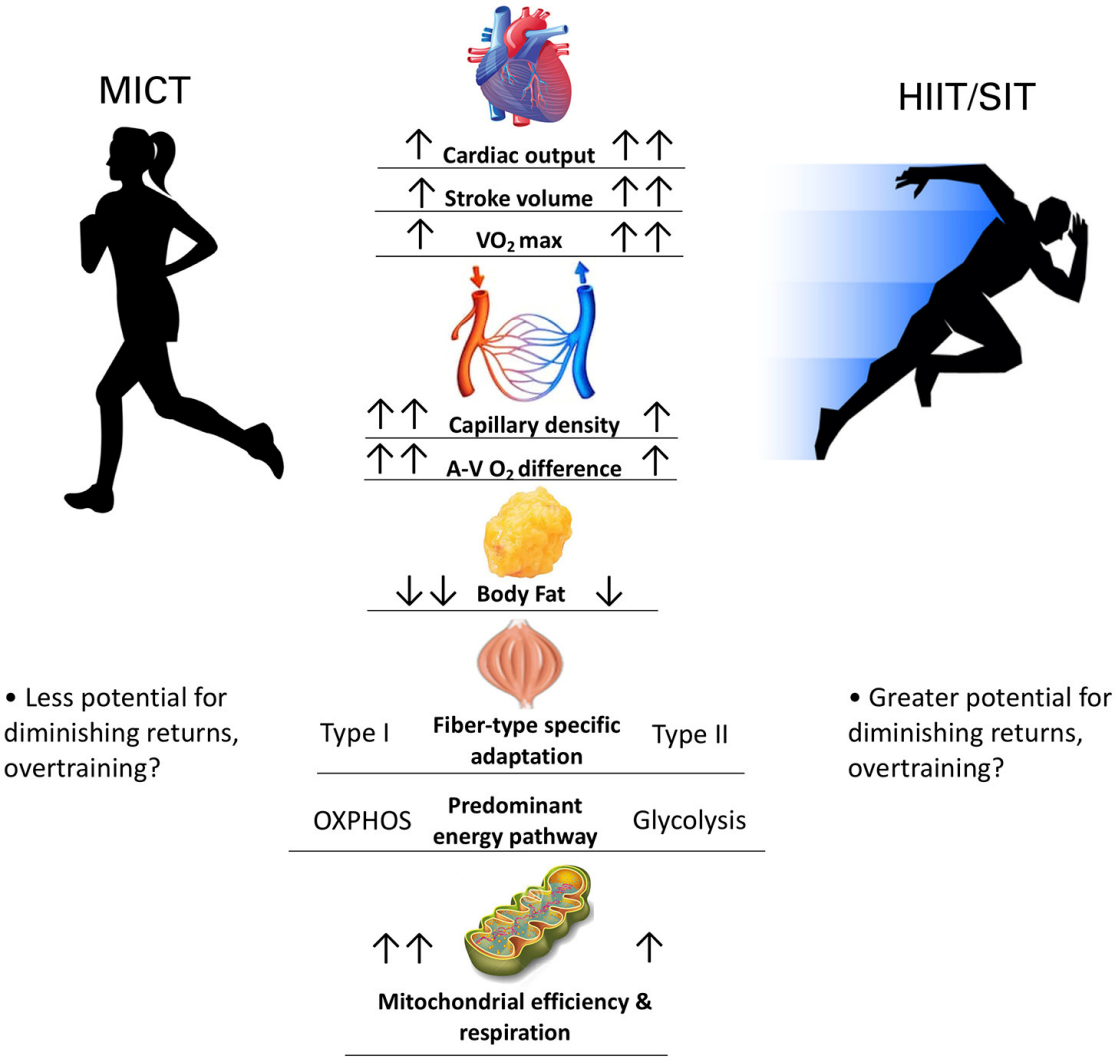
Comparing physiological adaptations between MICT and HIIT/SIT. Two arrows denotes greater magnitude of adaptation.

A broad overview of work in this field suggests that while HIIT provides robust a robust stimulus for central cardiovascular adaptations and metabolic stress (Gorostiaga et al., 1991; Macinnis and Gibala, 2017), MICT may preferentially target peripheral adaptations aimed at improving muscular oxygen extraction and metabolic efficiency (Gorostiaga et al., 1991; Beere et al., 1999; Daussin et al., 2008) (Figure 1). Indeed, a volume-matched comparison study between MICT and SIT showed that MICT conferred greater mitochondrial adaptation and lower blood lactate at the same relative work rate, whereas SIT demonstrated greater VO_2_max and peak power increases (Gorostiaga et al., 1991). The magnitude and rate of adaptations likely differs between HIIT and MICT training, but the heterogeneity (e.g., sample size, subject characteristics, training protocols) and lack of available comparison studies precludes definitive conclusions (Macinnis and Gibala, 2017). Moreover, longer-term training studies (>1yr) are needed to validate the safety and efficacy of long-term HIIT, as it is currently unclear how physiological adaptations to HIIT vs. MICT would manifest over more than the typical 8–12 weeks employed in conventional training studies. Extrapolating these data to suggest HIIT as the superior exercise mode, or as a [retracted] 2019 meta-analysis coined it: a “magic bullet” (Viana et al., 2019), is premature and unjustified given the current literature.

## Discussion

A one-size-fits-all approach is rarely the case in physiology. Further, a training distribution of >20% higher intensity training at the expense of MICT may cause autonomic disruption and diminishing performance returns (Stoggl and Sperlich, 2015). While this work examined elite endurance athletes who are not representative of the general population, a regimen consisting of only HIIT/SIT could promote overtraining pathology and stagnate the training process along with persistent complex I bypass which could prove to be undesirable resulting in a curtailment of performance/health adaptations. Thus, appreciable amounts MICT appears to be a necessary component of training, supported by the proposed volume-dependence of mitochondrial adaptation to exercise (Bishop et al., 2019b). If improved health is the desired outcome, adopting a pyramidal training intensity distribution similar that of elite athletes (Stoggl and Sperlich, 2015) with varying intensities may likely the preferred approach to promote sustainability and to reap all the cardiometabolic benefits of high and low intensity training.

If exercise is truly to be treated as a medicine, a rigorous acknowledgment of the dose is warranted. The dose should be chosen with the intent of optimizing the intended goal and a firm understanding of training adaptations allows informed decision making by clinicians, coaches, and athletes. To quote from Montero and Lundby: “The ultimate goal of any area of physiology is to discover the fundamentals of how a given function works, thus empowering to modify outcomes as desired” (Montero and Lundby, 2018). The work of Nilsson et al. discovered a potential fundamental aspect of exercise physiology. HIIT is indeed a time efficient strategy for rapid health improvements by providing a potent stimulus to heart and skeletal muscle and should undoubtedly be incorporated into exercise programs, although practitioners need to be cautious of diminishing returns, potential for overtraining, and not to neglect the distinct benefits obtained with MICT. For athletes, it comes as no surprise that HIIT should be prioritized for events where maximal aerobic capacity will be rate-limiting (i.e., middle distance running). Meanwhile, for more prolonged events (i.e., marathon, triathlon), substrate efficiency is rate-limiting and thus substantial MICT is warranted. Coaches, athletes, and physiologists have long understood the importance of training specificity without complex laboratory techniques but an appreciation for unique intensity-dependent adaptations is important to optimize exercise prescription. As for clinical patients, particularly those with metabolic dysfunction (San-Millán and Brooks, 2018), a case could be made for employing ample MICT to restore maximal electron transport and respiration. Ironically, a pyramidal style of training with a MICT foundation, similar to what is observed in elite athletes, could be optimal for these individuals. San Milan and Brooks were able to demonstrate marked metabolic flexibility in professional cyclists compared to moderately active individuals and those with metabolic syndrome (San-Millán and Brooks, 2018). While enormous training volumes are required to achieve that level of metabolic performance and are not feasible for the average individual, constructing modified exercise programs modeled similarly to that of endurance athletes will surely benefit those plagued with chronic disease. Additionally, a 12-week training study in inactive overweight adults demonstrated MICT to result in greater decreases in percentage of trunk and android fat compared to HIIT (Keating et al., 2014), whereas a 16-week study in MetS patients showed MICT reduced triglycerides more than HIIT, but both MICT and 4 × 4-min HIIT resulted in a similar lowering of overall MetS risk factors (Morales-Palomo et al., 2019). This is not to discount the known value of HIIT for diabetics (Cassidy et al., 2016), or cardiovascular disease (Angadi et al., 2015), but to emphasize the potential metabolic significance of incorporating ample MICT for an appropriate balance of training that encompasses the beneficial adaptations associated with both exercise “doses.” Future research should expand on the concept of complex I bypass to determine the relevance of CI_max_ to health and performance, and if it can indeed be used along with other factors to optimize exercise prescription.

## Author Contributions

SL drafted the manuscript. SL and GG contributed equally on editing the manuscript. All authors contributed to the article and approved the submitted version.

## Conflict of Interest

The authors declare that the research was conducted in the absence of any commercial or financial relationships that could be construed as a potential conflict of interest.
